# A Study on the Development and Validation of an Intergenerational Solidarity Measuring Tool Using Mixed Methods

**DOI:** 10.1093/geroni/igab046.1173

**Published:** 2021-12-17

**Authors:** Joosuk Chae, Seok In Nam

**Affiliations:** 1 Comprehensive Support Center for Seniors Living Alone, Seoul, Seoul-t'ukpyolsi, Republic of Korea; 2 Yonsei University, Seoul, Seoul-t'ukpyolsi, Republic of Korea

## Abstract

Intergenerational conflicts caused by rapid socioeconomic changes have highlighted the importance of strengthening intergenerational solidarity, emphasizing the necessity of tool designed to measure intergenerational solidarity. This study developed a standardized intergenerational solidarity measurement tool using mixed methods. In the qualitative research stage, 27 main survey questions were derived through literature research, in-depth interviews, and content validity verification. In the quantitative research stage, based on the results of a survey of 1,109 adults, both exploratory and confirmatory factor analyses of the questions were conducted, and the validity of the questions was confirmed. The analysis results were used to develop a 10-item measurement tool consisting of two factors: “recognition of intergenerational solidarity in the family” and “recognition of social intergenerational solidarity.” This study is the first attempt to develop a standardized measure of intergenerational solidarity, and it can be used for nationwide panel surveys in academic and policy research.

